# New methodology of TMB assessment from tissue and liquid biopsy in NSCLC

**DOI:** 10.1371/journal.pone.0275121

**Published:** 2022-09-26

**Authors:** Ľudmila Křížová, Markéta Šafaříková, Marta Kalousová, Lucie Pfeiferová, Aleš Antonín Kuběna, Michal Vočka, Jan Ulrych, Věra Franková, Luboš Petruželka, Tomáš Zima, David Feltl

**Affiliations:** 1 Department of Oncology, General University Hospital in Prague and First Faculty of Medicine, Charles University, Prague, Czech Republic; 2 Institute of Medical Biochemistry and Laboratory Diagnostics, General University Hospital in Prague and First Faculty of Medicine, Charles University, Prague, Czech Republic; 3 First Department of Surgery, Department of Abdominal, Thoracic Surgery and Traumatology, First Faculty of Medicine, Charles University and General University Hospital, Prague, Czech Republic; 4 Department of Paediatrics and Inherited Metabolic Disorders, General University Hospital and First Faculty of Medicine, Charles University, Prague, Czech Republic; Goethe University Hospital Frankfurt, GERMANY

## Abstract

Immunotherapy has dramatically influenced and changed therapeutical approach in non-small cell lung cancer (NSCLC) in recent five years. Even though we can reach long-term response to this treatment in approximately 20% of patients with NSCLC, we are still not able to identify this cohort of patients based on predictive biomarkers. In our study we have focused on tumor mutation burden (TMB), one of the potential biomarkers which could predict effectiveness of check-point inhibitors, but has several limitations, especially in multiple approaches to TMB quantification and ununiform threshold. We determined the value of TMB in tumor tissue (tTMB) and blood (bTMB) in 20 patients with early stage NSCLC using original custom gene panel LMB_TMB1. We evaluated various possibilities of TMB calculation and concluded that TMB should be counted from both somatic non-synonymous and synonymous mutations. Considering various factors, we established cut-offs of tTMB in/excluding *HLA* genes as ≥22 mut/Mb and 12 mut/Mb respectively, and cut-offs of bTMB were defined as ≥21 mut/Mb and ≥5 mut/Mb, respectively. We also observed trend in correlation of somatic mutations in *HLA* genes with overall survival of patients.

## Introduction

Immunotherapy have dramatically changed clinical oncology in the last five years. Although immunotherapy is already the standard of care in many oncological diagnoses and many clinical trials with new drugs are underway, we still do not know a reliable predictive biomarker that can identify those less than 20% of patients with non-small cell lung carcinoma (NSCLC) who respond to this treatment and have a long-term response [1]. Programmed death-ligand 1 (PD-L1) is a commonly used predictive biomarker in immuno-oncology–in clinical practice or in clinical trials to stratify patients. But PD-L1 as biomarker has fundamental problem which is the limited correlation between PD-L1 expression and response to immunotherapy [2].

Tumor mutation burden (TMB) is one of promising immune-oncological biomarkers, which is usually defined as the total amount of somatic coding mutations in a tumor DNA [3]. Values of TMB differ significantly across different cancer types. The highest TMB is in tumors associated with DNA environmental damage (tobacco smoke, UV light) such as lung cancer and melanoma [4, 5]. Tumors with high rate of mutations most likely express neoantigenes recognized as non-self by the immune system. Thus, after the stimulation of immune system by immunotherapy there is higher probability of the response to the treatment if TMB is high, and immune system reacts to various neoantigenes on tumor cells [6]. This hypothesis of association between presence of high amount of neoantigens and clinical response to immunotherapy was proved in many studies [3, 7, 8].

Recently published studies have shown certain correlation between accumulation of somatic mutations in human leukocyte antigen (*HLA*) genes and occurrence of high level of tumor neoantigenes. HLA system consists of cell surface molecules specialized to present antigenic peptides, which provide the ability of human immune system to recognize infected or cancerous cells. It has been implicated that *HLA* gene mutations could play a role in the mechanism of immune evasion during tumorigenesis [9]. McGranahan et al. confirmed loss of heterozygosity (LOH) in *HLA* genes in 40% of NSCLC and associated *HLA* LOH with high subclonal neoantigen burden [10]. Castro et al. concluded in their work that somatic *HLA* mutations were associated with higher overall mutation burden. Especially in tumors with microsatellite instability *HLA* mutations were highly enriched [11].

Using TMB as predictive biomarker has some limitations. Even though TMB has been used in many clinical studies, there is not any internationally determine threshold, from how many mutations to the megabase (Mb) is the sample considered positive. On the market, there are several different platforms and laboratories use different approaches to assess TMB and therefore results from different studies are incomparable and non-reproducible [12].

Fancello et al. have demonstrated large differences between various platforms for TMB quantification. Discrepancies occur at all levels of TMB assessment from methodology to the use of bioinformatics pipelines. They concluded that the lack of harmonization between different panel-based TMB quantifications is the main limitation of using TMB in routine practice. There is also no tool on to convert TMB estimates across different panels and of robust predictive cut-offs [3].

Rapid advances in molecular genetic methods have allowed to determine TMB from circulating tumor DNA (ctDNA) in the blood, this new biomarker is referred as blood-based TMB (bTMB) [13]. Gandara et al. have confirmed relatively high concordance of the tissue TMB (tTMB) results and bTMB [14].

The aims of our pilot project were firstly, to determine the value of tTMB in 20 patients with early stage of NSCLC and subsequently set cut-offs for tTMB positivity. Secondly, to implement tTMB workflow on bTMB in these patients and correlate results obtained from blood and tissue. Finally, to establish the utility of somatic mutations in *HLA* genes in TMB quantification. In our study we wanted to establish a functional methodology of TMB quantification in both tumor tissue and liquid biopsy. For TMB calculation we used our original custom gene panel LMB_TMB1. The results of tTMB and bTMB were also correlated with clinical data.

## Material and methods

### Patients

We enrolled 20 patients with NSCLC. All patients had localised operable lung tumor in early stage. We also collected different clinical data, see Table 1. The observation of patients and analysis of retrospective clinical data in our study was done from the date of diagnosis until the date of death or until the 30th of June 2020 (median of the time of observation was 25 months). The study has been approved by Ethics Committee of General University Hospital, Prague (ethical approval number 1061/19 S-IV). All enrolled patients have signed written informed consent with participation.

**Table 1:** Clinical data of patients included in our study.

Characteristic	Group	Number/Value
Carcinoma type (histologically)	Adenocarcinoma	11
	Spinocellular	9
NSCLC stage by TMN classification (8^th^ edition)	IA	1
	IB	5
	IIA	6
	IIB	4
	IIIA	4
Average age at the time of diagnosis (years)		66.6 ± 7.4
Median age at the time of diagnosis (years)		67.5
Sex	Male	13
	Female	7
Smoking	Yes	18
	No	2
*EGFR* status (established in 10 patients)*	Positive	2
	Negative	8
After surgical resection	Adjuvant chemotherapy	6
	Remission	10
	Death	7
	ND	3

* The *EGFR* status analysis was performed using kit EGFR XL StripAssay® (ViennaLab, Austria).

ND—These patients did not continue at follow-up after the surgery in our hospital.

### Sample collecting and DNA isolation

All samples (genomic DNA from blood cells, plasma, serum and tumor tissues) included in this study were collected and stored via Bank of Biological Material of First faculty of Medicine (BBM, http://biobanka.lf1.cuni.cz/), we used the samples for purpose of our study in accordance with the guidelines of the BBM and the informed consent of the patients. The sampling (blood and tissue) was done on operation day. Blood samples for blood cells and plasma were collected into tubes containing ethylenediaminetetraacetic acid (EDTA) and samples for serum were collected into tubes without anticoagulant. To obtain blood cells, plasma and serum, the collected blood samples were centrifuged for 15 min at 900 g and RT and almost all plasma and serum were transferred into new tubes. Aliquots with plasma and serum were promptly stored at -80°C; blood cell samples were stored in the refrigerator (2–8°C) until DNA extractions were performed. Genomic DNA (gDNA) from blood cells was extracted using manual salting-out procedure described by Miller et al. [15] with slight modification [16] These samples were diluted with Tris-EDTA (TE) buffer to the maximal concentration 300 ng/μL. All gDNA samples were quantified using absorbance at 260 nm (NanoDropTM 1000 Spectrophotometer, ThermoFisher Scientific, USA) and purity was analyzed by calculation of absorbance ratio 260/280 nm. All values were in the range 1.8–2.0 that is considered to represent pure DNA. The extracted gDNA was stored at -80°C. Native tumor tissues were aliquoted after samples for patient diagnosis had been collected. These aliquots were subsequently stored in liquid nitrogen.

Tumor tissue gDNA and circulating tumor DNA (ctDNA) were isolated from samples obtained from the collection of our biobank. Tumor tissue gDNA was isolated manually using QIAamp DNA Mini Kit (Qiagen, Germany) according to manufacturer’s instructions in the Laboratory of Molecular Pathology, Institute of Pathology, First Faculty of Medicine, Charles University and General University Hospital in Prague. The obtained yields were quantified using absorbance at 260 nm as described above and their integrity was analyzed using Genomic DNA Screen Tape and Reagents on TapeStation 4200 (Agilent, USA). All values of absorbance ratio 260/280 nm and DNA integrity number (DIN) except one were in the range 1.8–2.0 and 5.3–7.1, respectively (values of one tumor sample were 2.06 and 4.6, respectively). Samples of gDNA were stored at -80°C until analysis.

At first, we randomly selected 5 patients to test more suitable source of liquid biopsy. Their plasma and serum underwent second centrifugation (16 000 g for 15 minutes and 4°C) just before the isolation and ctDNA was extracted using QIAamp Circulating Nucleic Acid Kit (Qiagen, Germany) with QIAvac 24 plus system (Qiagen, Germany) according to manufacturer’s instructions. The quality and quantity of isolated ctDNA was analyzed using High Sensitivity D1000 Screen Tape and Reagents on TapeStation 4200 (Agilent, USA) according to manufacturer’s instructions. All ctDNA was subsequently purified using Agencourt AMPure XP Kit (Beckman Coulter, USA) according to manufacturer’s instructions and the quality of samples was checked again. Since only a small amount of ctDNA was obtained from plasma, we decided to use only serum ctDNA for NGS library preparation. The remaining sera were processed in the same way with only one difference. After purification, Cell-free DNA Screen Tape and Reagents were used for quality and quantity control. The proportion of cfDNA in these sera was > 90%. Samples of ctDNA were subsequently stored either in the refrigerator (2–8°C) in case of immediate NGS library preparation or in -20°C in case of delay in NGS library preparation.

### NGS library preparation and sequencing

For purpose of this study, we have designed our original custom gene panel (LMB_TMB1) containing coding exons of 313 genes, see Table 2. All NGS libraries were prepared using SureSelectXT HS Reagent Kit (Agilent, USA) with SureSelectXT Custom 0.5–2.9Mb (Agilent, USA). Blood cell and tumor tissue gDNA was fragmented using SureSelectXT HS Enzymatic Fragmentation Kit (Agilent, USA). The quantity of enter gDNA and NGS libraries during and after preparation was checked using dsDNA HS Assay Kit with Qubit fluorometer (Invitrogen, USA) and quality of NGS libraries was checked using High Sensitivity D1000 Screen Tape and Reagents on TapeStation 4200 (Agilent, USA). All prepared NGS libraries were sequenced on NextSeq 500 (Illumina, USA), using NextSeq 500/550 Mid or High Output Kit v2.5, 300 cycles (Illumina, USA). All commercial kits were used according to manufacturer’s instructions.

**Table 2:** List of genes included in our panel LMB_TMB1.

*ABL1*, *AGER*, *AKT1*, *AKT2*, *AKT3*, *ALK*, *AMER1*, *APC*, *AR*, *ARAF*, *ARID1A*, *ASXL1*, *ATM*, *ATR*, *ATRX*, *AURKA*, *AURKB*, *AXIN1*, *AXL*, *BAP1*, *BARD1*, *BCL2*, *BCL3*, *BCL6*, *BCOR*, *BCORL1*, *BRAF*, *BRCA1*, *BRCA2*, *BRD4*, *BRIP1*, *BTK*, *C11ORF30*, *CARD11*, *CASP8*, *CBFB*, *CBL*, *CCND1*, *CCND2*, *CCND3*, *CCNE1*, *CD274*, *CD79A*, *CD79B*, *CDC73*, *CDH1*, *CDK12*, *CDK4*, *CDK6*, *CDK8*, *CDKN1A*, *CDKN1B*, *CDKN2A*, *CDKN2B*, *CDKN2C*, *CEBPA*, *CIC*, *CREB1*, *CREBBP*, *CRKL*, *CSF1R*, *CTCF*, *CTNNA1*, *CTNNB1*, *CXCR4*, *CYLD*, *CYP17A1*, *CYP2A7*, *DAXX*, *DCC*, *DDR1*, *DDR2*, *DICER1*, *DNMT3A*, *DOT1L*, *EGFR*, *EP300*, *EPHA3*, *ERBB2*, *ERBB3*, *ERBB4*, *ERG*, *ERRFI1*, *ESR1*, *EZH2*, *FAM175A*, *FAM46C*, *FANCA*, *FANCB*, *FANCC*, *FANCG*, *FANCI*, *FANCL*, *FANCM*, *FAS*, *FBXW7*, *FGF10*, *FGF12*, *FGF14*, *FGF19*, *FGF2*, *FGF23*, *FGF3*, *FGF4*, *FGF6*, *FGFBP1*, *FGFR1*, *FGFR2*, *FGFR3*, *FGFR4*, *FH*, *FLCN*, *FLT1*, *FLT3*, *FLT4*, *FOXL2*, *FUBP1*, *G6PD*, *GABRA6*, *GATA1*, *GATA2*, *GATA3*, *GATA4*, *GATA6*, *GC*, *GLO1*, *GNA11*, *GNA13*, *GNAQ*, *GNAS*, *GPC3*, *GRM3*, *H3F3A*, *HGF*, *HIF1A*, *HLA-A*, *HLA-B*, *HLA-C*, *HLA-DPA1*, *HLA-DPB1*, *HLA-DQA1*, *HLA-DQB1*, *HLA-DRA*, *HLA-DRB1*, *HMGB1*, *HNF1A*, *HNF1B*, *HRAS*, *HSD3B1*, *HSP90AA1*, *HSP90AB1*, *CHEK1*, *CHEK2*, *IDH1*, *IDH2*, *IFNGR1*, *IFNGR2*, *IGF1R*, *IGF2R*, *IKBKB*, *IKBKE*, *IKZF1*, *IL1B*, *IL1RN*, *IL6*, *INPP4B*, *IRF1*, *IRF2*, *IRF4*, *IRS2*, *JAK1*, *JAK2*, *JAK3*, *JUN*, *KDM5A*, *KDM5C*, *KDM6A*, *KDR*, *KEAP1*, *KIT*, *KLHL6*, *KMT2A*, *KMT2D*, *KRAS*, *LYN*, *MAP2K1*, *MAP2K2*, *MAP2K4*, *MAP3K1*, *MAP3K13*, *MAPK1*, *MCL1*, *MDM2*, *MDM4*, *MED12*, *MEF2B*, *MEN1*, *MET*, *MITF*, *MLH1*, *MMP2*, *MMP8*, *MMP9*, *MPL*, *MRE11A*, *MSH2*, *MSH6*, *MST1R*, *MTOR*, *MUC1*, *MUTYH*, *MYC*, *MYCL*, *MYCN*, *MYD88*, *NF1*, *NF2*, *NFE2L2*, *NFKB1*, *NFKB2*, *NFKBIA*, *NLRC5*, *NOTCH1*, *NOTCH2*, *NOTCH3*, *NPM1*, *NRAS*, *NTRK1*, *NTRK2*, *NTRK3*, *PALB2*, *PAPPA*, *PAPPA2*, *PARK2*, *PARP1*, *PARP2*, *PARP3*, *PARP4*, *PAX5*, *PBRM1*, *PDCD1LG2*, *PDF*, *PDGFRA*, *PDGFRB*, *PDK1*, *PGF*, *PIK3C2B*, *PIK3CA*, *PIK3CB*, *PIK3R1*, *PMS2*, *POLD1*, *POLE*, *PRDM1*, *PRKAR1A*, *PTEN*, *PTCH1*, *PTPN11*, *RAC1*, *RAD51*, *RAF1*, *RARA*, *RB1*, *RBM10*, *RBP4*, *RET*, *RFC2*, *RICTOR*, *RNF2*, *RNF43*, *ROS1*, *RPTOR*, *S100A12*, *SDHA*, *SDHB*, *SDHC*, *SDHD*, *SETD2*, *SF3B1*, *SMAD4*, *SMARCA4*, *SMARCB1*, *SMO*, *SOCS1*, *SOX2*, *SOX9*, *SPEN*, *SPOP*, *SRC*, *STAG2*, *STAT3*, *STK11*, *STRA6*, *SUFU*, *SYK*, *TBX3*, *TEK*, *TET2*, *TGFBR2*, *TKTL1*, *TLR2*, *TLR4*, *TNFAIP3*, *TNFRSF14*, *TP53*, *TSC1*, *TSC2*, *U2AF1*, *VDR*, *VEGFA*, *WT1*, *XPO1*, *ZNF217*

### Data analysis

First, the raw data in BCL format downloaded from sequencer were converted to FASTQ format using bcl2fastq Conversion Software v2.20 (Illumina, USA). All other NGS data analyses were performed using commercial software CLC Genomic Workbench 12 (Qiagen, Germany). Reads were aligned to Homo sapiens reference genome hg19 and data were subsequently annotated using quality score, minimum number of reads and coverage. Databases clinvar_20171029_hg19 and dbsnp_v150_hg19 were used for variant annotation and the filtration workflow was developed to obtain variants suitable to be included in tumor mutational burden (TMB) count.

### TMB calculation and statistical analysis

For assessment of the level of both tTMB and bTMB we used four different approaches. In two approaches, we included all genes of our panel LMB_TMB1, one containing all identified mutations and the other only non-synonymous mutations. In two calculations, we excluded *HLA* genes because they are not usually included in gene panels for TMB assessment. For these calculations we applied the same two approaches as described above (all mutations vs. only non-synonymous mutations).

Clinical data were corelated with the results of TMB using the program STATISTICA 12 (Statsoft CR s.r.o., Czech Republic) and tests Mann-Whitney U Test and Kruskal-Wallis ANOVA.

Survival analysis was performed using Wolfram Mathematica 11.3 (Wolfram Research, UK). Cox regression was used to evaluate quantitative survival predictors. Since the number of events usually has the statistical distribution near to Poisson, before evaluation, we have adapted them by the Box-Cox logarithmic power transformation.

All results were considered statistically significant at p<0.05.

## Results

The Spearman analysis of all tTMB calculations showed very strong correlation between all calculation approaches (R>0.85, p<0.0001). The same analysis in bTMB showed also a strong statistically significant relationship between all calculations (R>0.56, p<0.01) except one value. The relationship between non-synonymous mutation approach including *HLA* genes and all mutation approach excluding *HLA* genes was slightly weaker but still very significant (R>0.52, p = 0.018).

The correlation analysis between same approaches of tTMB and bTMB calculations showed the relationship only between all mutation TMBs including *HLA* genes (R = 0.58, p = 0.007) and non-synonymous mutation TMBs including *HLA* genes (R = 0.52, p = 0.018). All mutation TMBs excluding *HLA* genes did not show statistically significant correlation (R = 0.43, p = 0.06), however, there was a certain association trend.

Based on correlation results mentioned above, the tTMB/bTMB positivity cut-offs including and excluding *HLA* genes were defined as ≥22/21 mut/Mb (R = 0.76, p<0.0001) and ≥12/5 mut/Mb, respectively (R = 0.64, p = 0.002). The correlation between defined tTMB cut-offs is very strong (R = 0.76, p<0.0001) and the relationship between bTMB cut-offs is even more evident (R = 0.84, p<0.0001). All TMB values are summarized in Table 3.

**Table 3:** Results of tTMB and bTMB using different approach of TMB calculation and our gene panel LMB_TMB1.

	Histology	tTMB all^a)^	bTMB all^b)^	tTMB non-synomyous all^c)^	bTMB non-synomyous all^d)^	tTMB nonHLA^e)^	bTMB nonHLA^f)^	tTMB non-synomyous nonHLA^g)^	bTMB non-synomyous nonHLA^h)^
1	Aca	**52**	**35**	38	28	**44**	**8**	31	5
2	Aca	**44**	**56**	35	41	**33**	**41**	24	31
3	Aca	**41**	**43**	27	28	**21**	**23**	14	14
4	Aca	(17)	(20)	14	14	**14**	**5**	11	5
5	Aca	**53**	**30**	41	22	**41**	**11**	33	8
6	Aca	(6)	(17)	4	11	(5)	(4)	3	3
7	Aca	**27**	**42**	20	28	**21**	**11**	16	8
8	Aca	**30**	**46**	25	37	10^x^	6^x^	8	5
9	Aca	(16)	(10)	15	7	(11)	(4)	11	4
10	Aca	11^x^	33^x^	5	21	6^x^	9^x^	5	6
11	Aca	**77**	**21**	58	9	**76**	**8**	58	4
12	SCC	**27**	**22**	19	20	**14**	**6**	10	6
13	SCC	**35**	**30**	25	19	**23**	**6**	19	3
14	SCC	**60**	**69**	48	51	**35**	**19**	28	14
15	SCC	(21)	(10)	19	5	(9)	(4)	8	3
16	SCC	**53**	**53**	36	37	**23**	**6**	16	4
17	SCC	**23**	**35**	20	25	**15**	**5**	11	4
18	SCC	19^x^	40^x^	16	31	8^x^	11^x^	6	9
19	SCC	**26**	**21**	16	12	**15**	**9**	9	5
20	SCC	**54**	**57**	44	42	**24**	**5**	21	5

Aca = adenocarcinoma; SCC = spinocellular carcinoma.

Bold = Matching TMB positivity (= high TMB) in tissue and serum using all mutation calculation in/excluding HLA genes (tissue ≥22/12 mut/Mb and serum ≥21/5 mut/Mb).

() = Matching TMB negativity (= low TMB) in tissue and serum using all mutation calculation in/excluding HLA genes (tissue <22/12 mut/Mb and serum <21/5 mut/Mb).

^x^ Mismatch in TMB positivity/negativity between tTMB and bTMB in the same TMB calculation approach with defined cut-offs.

^a)^ tTMB all = all tissue somatic mutations in all genes.

^b)^ bTMB all = all liquid biopsy somatic mutations in all genes.

^c)^ tTMB non-synonymous all = all tissue non-synonymous somatic mutations in all genes.

^d)^ bTMB non-synonymous all = all liquid biopsy non-synonymous somatic mutations in all genes.

^e)^ tTMB nonHLA = all tissue somatic mutations in all genes except HLA genes.

^f)^ bTMB nonHLA = all liquid somatic mutations in all genes except HLA genes.

^g)^ tTMB non-synonymous nonHLA = all tissue non-synonymous somatic mutations in all genes except HLA genes.

^h)^ bTMB non-synonymous nonHLA = all liquid biopsy non-synonymous somatic mutations in all genes except HLA genes.

Due to the small sample size in our project, we did not observe any subgroup of patients (according to histology, TNM, clinical stage, gender and age) in whom the tTMB or bTMB were statistically significantly increased. No statistically significant results were also found in the comparison of clinical subgroups mentioned above with TMBs divided into categories “high” tTMB (in/excluding *HLA* genes) ≥22/12 mut/Mb, “low” tTMB <22/12 mut/Mb (in/excluding HLA genes); and “high” bTMB ≥21/5 mut/Mb (in/excluding HLA genes), “low” bTMB <21/5 mut/Mb (in/excluding *HLA* genes). The effect of smoking could not be statistically evaluated (18 smokers vs. 2 non-smokers) as well as the effect of *EGFR* mutations (2 positive, 8 negative, 10 patients without *EGFR* mutation status).

The interesting results were obtained by comparing the number of HLA mutations identified in tumor tissue and serum. The *HLA* mutations were divided into three categories: “all”, “non-synonymous” and “synonymous”. The tissue-serum correlations were showed in all mutation category (R = 0.54, p = 0.015) and non-synonymous category (R = 0.57, p = 0.008).

We also wanted to evaluate both tTMB and bTMB in terms of survival. Tumor tissue and serum TMB values obtained by our gene panel and calculated according to the all-mutation approach excluding *HLA* genes, approx. 1 Mb, were statistically analysed with Box-Cox log transformation n→Log[n] (n = TMB value) and did not show any significance for patients´ survival. Subsequently, we focused on *HLA* genes in which relatively high number of mutations in a small region was detected. Due to the quite different sequenced lengths between *HLA* genes (0.01 Mb) and the rest of the panel (0.80 Mb) which could skew results in case of using TMB calculation with an approximation to 1 Mb, for further statistical evaluation we preferred the absolute number of exon somatic mutations to TMB value. All identified mutations were divided into four groups as potential survival predictors: synonymous mutations in *HLA* genes, non-synonymous mutations in *HLA* genes, synonymous mutations in the rest of the gene panel and non-synonymous mutations in the rest of the gene panel. Again, Box-Cox power logarithmic transformation was performed, namely n→Log[1+n], n = number of mutations. As an alone predictor, none of the numbers of mutations had impact on survival. However, all models including both synonymous and non-synonymous *HLA* mutations in tissue suggested the trend of importance of *HLA* mutations in term of patients´ survival but in the opposite direction: non-synonymous mutations decreased the risk of death (p≤0.05) while synonymous *HLA* mutations increased it (p≤0.08). The results of the same analysis with serum *HLA* mutations were not statistically significant.

## Discussion

We have decided to use NSCLC as model disease for tTMB and bTMB assessment for two reasons. First, immunotherapy is standard of care in patients with advanced NSCLC and it starts to appear in both neoadjuvant and adjuvant settings. Second, to acquire the tissue sample of lung tumor is sometimes challenging and thus using liquid biopsy might be elegant option how to analyse the tumor DNA. The main limitations of TMB assessment from liquid biopsy is the low amount of available ctDNA, which affects sensitivity and concerns that ctDNA is more associated with metastases than with primary tumors [17–19]. To avoid this issue we enrolled patients with localised NSCLC, thus the bTMB result won’t be affected by the presence of metastases. TMB in early-stage lung cancer is not often studied, and results of our work would bring a new information about this cohort of patients. In our work both tissue and blood collecting were performed on the same day and therefore the results should not be affected by mutations developed in time.

The predictive and prognostic value of TMB, despite the effort of scientific community, was not clearly established. There is still lack of harmonization between different assessed platforms, which might be the cause of low reproductivity of results across publications resulting in a series of contradictory and hardly evaluated data. For instance, based on the data from Kenote-158 trial, FDA (Food and Drug administration) approved pembrolizumab for all solid malignancies with tTMB high status. In this trial pembrolizumab was tested on more than a thousand patients with different malignancies. Results showed association between TMB-high and both higher objective response rate (ORR) and better progression-free survival (PFS) [20]. On the other hand, analysis of 34 clinical trials containing 7700 patients with various cancers treated with immunotherapy, demonstrated that 79% of included trials did not report any statistically significant association between TMB and overall survival (OS) [21]. In NSCLC the situation is not clearer. Even though metanalysis of five randomized controlled phase III studies observed benefit in ORR, PFS and OS in favour of immunotherapy agents in the TMB-high NSCLC population [22]. Many trials did not prove predictive value of TMB in subanalysis and most of this data was not even properly published resulting in impossibility of subsequent evaluation. Until now the effectiveness of immunotherapy in correlation with TMB status was studied inclusively in a palliative indication. But immunotherapy especially in combination with chemotherapy is tested in both adjuvant and neoadjuvant settings in early NSCLC, and first positive phase III trials were already published [23, 24]. It is necessary to have reliable biomarker in group of potentially curable patients. However, in none currently published trial dealing with adjuvant or neoadjuvant treatment, TMB was used neither as a stratification factor nor a predictive biomarker. We can only assume that an additional analysis will be done. A very interesting new hypothesis was published recently suggesting non-linearity quantity of TMB [25]. Nevertheless, it was an isolated observation, and no other publications supported this finding.

New works focus more on prognostic potential of TMB. Robust analysis of impact of TMB on OS was done on 6035 patients with 20 primary solid cancer types from The Cancer Genome Atlas (TCGA) database. They observed tree different groups of cancer behaviour: TMB-Worse, TMB-Better and TMB-Similar group. TMB-Worse group contained 8 tumor types which in case of TMB high had worse prognosis. 6 tumor types from TMB-Better group had better prognosis if TMB high. Finally, in the last TMB-Similar group any differences in survival between TMB high and low patients were not noticed. This group consisted of 6 tumor types including NSCLC [26]. In different publication TMB high status was associated with longer overall survival and disease-free survival in early-stage NSCLC, based on analysis of more than 900 patients [27]. According to the literature above, TMB seems to be a very contradictory marker which would merit further study to harmonize and, above all, standardize its calculation.

A golden standard for tTMB assessment is the whole-exome sequencing (WES) [28], but the threshold for TMB positivity is not unequivocally established and varies across different studies [21, 29]. Given that the implementation and interpretation of WES in clinical practice is very demanding, gene panels have started to be used more for TMB analysis. In our study we designed custom gene panel consisted of 313 genes (LMB_TMB1) based on research of available published literature and panels (i.e. F1CDx, MSK-IMPACT, Illumina TSO500, Oncomine TML, QIAseq TMB) see Table 4 [30, 31]. As a standard method of calculation, we suggest including both synonymous and non-synonymous somatic mutations without excluding mutations or genes associated with cancer (e.g. *EGFR*). According to in-silico simulations, it is indicated that inclusion of all mutations, even if they may not directly contribute to immunogenicity, increases precision of TMB estimation when using targeted gene panels [32], as well as including both synonymous and non-synonymous mutations [33, 34]. Even though synonymous mutations are not directly involved in creating neoantigenes, their presence is a signal of mutational processes, which could result in non-synonymous mutations elsewhere in the genome [35].

**Table 4:** Comparison of currently used gene panels for tTMB calculation [30, 31] with our panel (LMB_TMB1).

Gene panel	Number of genes	Size region covered* (Mb)	TMB cut-off	Gene variants included in TMB calculation	FDA approved
F1CDx	324	0.8 Mb	10 mut/Mb	Somatic non-synonymous and synonymous mutations	Yes
MSK-IMPACT	468	1.14 Mb		Somatic non-synonymous mutations	Yes
Illumina TSO500	523	1.33 Mb	10 mut/Mb	Somatic non-synonymous and synonymous mutations	No
Oncomine TML	409	1.22 Mb		Somatic non-synonymous mutations	No
QIAseq TMB	486	1.33 Mb		Somatic non-synonymous mutations	No
LMB_TMB1	313	0.81/0.8 Mb**	22/12 mut/Mb**	Somatic non-synonymous and synonymous mutations***	No

* Coding region used to estimate TMB regardless of the size of the region assessed by the panel.

** The approach for TMB calculation including HLA genes / the approach for TMB calculation excluding HLA genes.

*** Non-synonymous mutations include SNPs and short insertions and deletions (indels).

We inclined to the opinion that every laboratory should determine its own threshold of TMB positivity according to genes included in the panel, in/excluding mutations or genes associated with cancer, types of mutations included in the TMB calculation (SNP vs. SNP plus indels and synonymous vs. synonymous plus non-synonymous) and analysed cancer type (cancer specific cut-offs). These thresholds should be subsequently validated. To determine the appropriate tTMB positivity threshold for our gene panel in NSCLC patients, we have considered following factors: i) differences in tTMB and bTMB correlation results, ii) types of included variants in TMB calculation and non-removing of mutations or genes associated with cancer, iii) gene included in our panel, iv) number of detected variants in *HLA* genes, v) studies mentioned below and vi) Fancello et al. review recommendations [3]. Finally, we decided to establish cut-offs according to in/excluding *HLA* genes. The cut-offs in/excluding *HLA* genes in tTMB were defined as ≥22 mut/Mb and 12 mut/Mb respectively. The 22 mut/Mb cut-off seems to be very clear. Due to the same correlation results, an equivocal zone of TMB values excluding *HLA* genes spans 12 mut/Mb to 13 mut/Mb.

Determining bTMB is more challenging than tTMB, less data is available, and approaches differ more between different works. As well as in tTMB assessment WES is golden standard of determination of bTMB, but some studies using gene panels were already done. A retrospective work confirmed correlation between tTMB and bTMB in patients with NSCLC included in OAK and POPLAR clinical trials, high TMB was associated with response to immunotherapy in both trials. The analysis was performed using FoundationOne [36]. Different work successfully correlated blood and tissue TMB results on 2000 NSCLC samples from Geneplus database. The same threshold 9 mut/Mb was used for all three types of TMB including single-region tTMB, multi-region TMB and bTMB [37]. Commercial bTMB platforms has already been available on market such as Guardant OMNI bTMB assay containing 500 genes with genome coverage of 2.1 Mb and already mentioned FoundationOne Medicine bTMB assay with 394 genes and 1.14 Mb coverage. Both platforms are comparable, and their results showed high correlation [38]. The cut-offs of bTMB high positivity have not yet been definitively established. In Guardant OMNI, bTMB threshold was defined as 16 mut/Mb but data from MYSTIC trial showed that cut-off 20 mut/Mb might be more clinically relevant in NSCLC [39]. FoundationOne bTMB assay cut-off was define as 14.5 mut/Mb (16 mut/1.1 Mb) [13].

To define the bTMB cut-off suitable for our gene panel we used a similar approach as mentioned above for tTMB thresholds. Our cut-offs in/excluding *HLA* genes in bTMB were defined as ≥21 mut/Mb and ≥5 mut/Mb, respectively. An equivocal zone of TMB values seems to span 18 mut/Mb to 21 mut/Mb and 5 mut/Mb to 6 mut/Mb, respectively. Because of the extremely low level of ctDNA in plasma measured in several of our samples, we decided to use serum instead of plasma.

Clear clinical relevance of bTMB is not yet establishment. Even though phase II trial B-F1RST demonstrated utility of bTMB as a predictive biomarker for atezolizumab monotherapy in advanced NSCLC, using cut-off 16 mut/Mb [40]. However, these findings were not confirmed by subsequent phase III trial BFAST cohort C (data not published) which compared chemotherapy versus atezolizumab monotherapy in bTMB high NSCLC population. In this trial, a typical crossover of the Kaplan Meier PFS and OS curves was observed, which is associated with the slow onset of the action of immunotherapy in monotherapy. Therefore, combined chemoimmunotherapy is now used in first line treatment for majority of NSCLC patients. The study did not show any increase in median PFS, but after more than four months of therapy, atezolizumab showed a significant improvement in PFS [41]. To conclude, bTMB might not be sufficient predictive biomarker to exclude chemotherapy in first line treatment and administered monotherapy of check point inhibitor. More data needs to be collected to apply this finding to clinical practise. Especially, it is necessary to determine what predictive value bTMB will have for other check point inhibitors in monotherapy.

We also observed interesting trend in correlation of somatic mutations in *HLA* genes with overall survival of patients. All models including both synonymous and non-synonymous *HLA* mutations suggested their importance. The non-synonymous mutations decreased the risk of death while synonymous *HLA* mutations increased it. The explanation might be in the amino acid change in relation with the mutation type. The amino acid change in non-synonymous mutations changes the antigen localized on cell surface which can activate patient’s immune system. The synonymous mutations do not influence the surface antigen and therefore the immune system does not react against the developing tumor. In addition, the correlation analysis of number of *HLA* mutations between tumor tissue and serum showed relationship in both all *HLA* mutations and non-synonymous mutations. Such results connecting somatic mutations in *HLA* genes in tumor cells and overall survival of patients were not yet published according to our knowledge. But clear conclusions cannot yet be drawn, because of the small number of patients. Only few articles focusing on this subject are available. Goodman et al. hypothesized that poor presentation of neoantigens by MHC-I explains why some tumors do not respond to immunotherapy independently to level of TMB [42]. Different article showed correlation between *HLA* genes loss of heterozygosity and level of TMB [10]. Shim et al. published new approach to TMB calculation, which consider the *HLA* genes mutation. This *HLA*-corrected TMB reconciles the observed disparity in relationships between TMB and immunotherapy responses [43]. Recent article described association of short PFS and OS with homozygosity at ≥1 *HLA-I* gene in patients with PDL1 high (>50%) advanced non-small cell lung cancer and receiving single-agent immunotherapy [44]. These various ambiguous conclusions of various articles suggest that there will certainly be some relationship between the response to immunotherapy, the TMB value, and changes in *HLA* genes. However, further research is needed.

## Conclusion

TMB is one of the important biomarkers of immunotherapy, but the determination of its value is not standardized. Therefore, we created a pilot project in which we wanted to set up a methodology and procedure for determining TMB from tissue and blood. Overall, it can be concluded that determining TMB using our gene panel is possible, and the results seem to be very promising for future use. Based on our results and the results of other recent works, we consider it more appropriate to use all mutations to calculate TMB. The threshold between TMB high and TMB low samples was in our work defined according to in/excluding *HLA* genes and differently for both tTMB and bTMB. When we used all somatic mutations for TMB calculation, the correlation between tTMB and bTMB was highly significant in approach including HLA genes and statistically unclear in approach excluding *HLA* genes. Subsequently, our results indicate the significance of mutations in *HLA* genes for patients´ prognosis and the need to consider both synonymous and non-synonymous mutations.

We must take into consideration the small number of analysed samples in our pilot study. The next step will be to perform a study with a larger number of patients with advanced and metastatic NSCLC, where it will be possible to evaluate a correlation between the effect of immunotherapy and the TMB value.

## Supporting information

S1 Data Somatic serum. (XLSX)

S2 Data Somatic tissue. (XLSX)
